# Muscle $\dot{V}O_{2}$‐power output nonlinearity in constant‐power, step‐incremental, and ramp‐incremental exercise: magnitude and underlying mechanisms

**DOI:** 10.14814/phy2.13915

**Published:** 2018-11-13

**Authors:** Bernard Korzeniewski

**Affiliations:** ^1^ BioSimulation Center Kraków Poland

**Keywords:** Constant‐power exercise, oxygen uptake kinetics, ramp‐incremental exercise, skeletal muscle, step‐incremental exercise

## Abstract

A computer model of the skeletal muscle bioenergetic system was used to simulate time courses of muscle oxygen consumption ($\dot{V}O_{2}$), cytosolic metabolite (ADP, PCr, P_i_, and ATP) concentrations, and pH during whole‐body constant‐power exercise (CPE) (6 min), step‐incremental exercise (SIE) (30 W/3 min), and slow (10 W/min), medium (30 W/min), and fast (50 W/min) ramp‐incremental exercise (RIE). Different ESA (each‐step activation) of oxidative phosphorylation (OXPHOS) intensity‐ATP usage activity relationships, representing different muscle fibers recruitment patterns, gave best agreement with experimental data for CPE, and for SIE and RIE. It was assumed that the muscle $\dot{V}O_{2}$‐power output (PO) nonlinearity is related to a time‐ and PO‐dependent increase in the additional ATP usage underlying the slow component of the $\dot{V}O_{2}$ on‐kinetics minus the increase in ATP supply by anaerobic glycolysis leading to a decrease in $\dot{V}O_{2}$. The muscle $\dot{V}O_{2}$‐PO relationship deviated upward (+) or downward (−) from linearity above critical power (CP), and the nonlinearity equaled +16% (CPE),+12% (SIE), +8% (slow RIE), +1% (moderate RIE), and −2% (fast RIE) at the end of exercise, in agreement with experimental data. During SIE and RIE, changes in PCr and P_i_ accelerated moderately above CP, while changes in ADP and pH accelerated significantly with time and PO. It is postulated that the intensity of the additional ATP usage minus ATP supply by anaerobic glycolysis determines the size of the muscle $\dot{V}O_{2}$‐PO nonlinearity. It is proposed that the extent of the additional ATP usage is proportional to the time integral of PO ‐ CP above CP.

## Introduction

Step‐incremental exercise (SIE) and ramp‐incremental exercise (RIE) (Whipp et al. 1981; Davies et al. 1982; Hansen et al. 1987; Zoladz et al. 1995; Rossiter 2011; Murgatroyd et al. 2014; Keir et al. 2016) are frequently applied in “pure” studies in human muscle physiology as well in medical diagnostics and exercise efficiency determination in sport sciences and practices.

In SIE and RIE power output (PO) can increase with different speed with time. For instance, PO can be elevated step‐wise by 30 W every 3 min (SIE, 30 W/3 min) or increase continuously with time at speed of 10 W per min (slow RIE, 10 W/min), 30 W per min (medium RIE, 30 W/min), or 50 W per min (fast RIE, 50 W/min).

Only whole‐body variables, such as pulmonary $\dot{V}O_{2}$ kinetics or blood lactate and blood pH, were measured or estimated in these kinds of exercise. On the other hand, such variables concerning directly the skeletal muscle bioenergetic system kinetic properties during exercise as muscle $\dot{V}O_{2}$, PCr, and cytosolic ADP, P_i_, ADP, and pH that can be measured for constant‐power exercise (CPE) in contracting calf (Allen et al. 1997) or in quadriceps during knee extension (Krustrup et al. 2009; Cannon et al. 2014) were not measured in whole‐body (e.g., cycling) SIE and RIE, mostly because of technical difficulties.

The nonlinearity in the pulmonary $\dot{V}O_{2}$‐PO relationship is observed in CPE, SIE, and RIE above critical power (CP) (see below). This nonlinearity in SIE was related to the slow component of the $\dot{V}O_{2}$ on‐kinetics (Zoladz et al. 1995, 1998). The nonlinearity in the pulmonary $\dot{V}O_{2}$‐PO relationship seems to be significantly greater in SIE than in medium RIE (24). In ramp‐incremental exercise (RIE) the nonlinearity of the pulmonary $\dot{V}O_{2}$‐PO relationship tends to be highest in slow RIE, medium in medium RIE, and lowest (negative, downward deviation) in fast RIE (Whipp et al. 1981; Davies et al. 1982; Hansen et al. 1987; Zoladz et al. 1995; Scheuermann et al. 2002; Rossiter 2011; Murgatroyd et al. 2014; Keir et al. 2016) (see Discussion). The mechanisms underlying the $\dot{V}O_{2}$‐PO nonlinearity and the differences in its size in various exercise modes are not fully understood.

The slow component of the $\dot{V}O_{2}$ on‐kinetics is an additional oxygen consumption above the principal (phase II) component of the $\dot{V}O_{2}$ on‐kinetics present in intensive exercise. The slow component appears just after exceeding the lactate threshold (LT), that is PO above which the blood lactate is elevated (Jones et al. 2011), and increases progressively with time when PO exceeds the so‐called critical power (CP) (Jones et al. 2010; Poole et al. 2016). CP, which corresponds to ~50–80% of the maximum oxygen consumption $\dot{V}O_{2\max}$ in healthy adults, is represented by the asymptote of the hyperbolic power versus time to exercise intolerance relationship (Poole et al. 1988; Jones et al. 2010). Therefore, CP is considered as physiological index of exercise intensity in humans above which no steady‐state in muscle metabolites such as PCr, P_i_, ADP_free_, and H^+^ can be maintained and the permanent slow component of the $\dot{V}O_{2}$ on‐kinetics appears (Poole et al. 1988; Jones et al. 2010; Rossiter 2011). It has been proposed that the progressively increasing in time slow component of the $\dot{V}O_{2}$ on‐kinetics is mainly caused by a linear increase with time of the additional ATP usage above CP (Korzeniewski and Rossiter 2015; Korzeniewski and Zoladz 2015). It has been demonstrated that majority (about 85%) of the magnitude of the pulmonary slow component originates within skeletal muscles (Poole et al. 1991).

The pulmonary $\dot{V}O_{2}$ on‐kinetics reproduces well the muscle $\dot{V}O_{2}$ on‐kinetics in pedaling of moderate intensity and knee‐extension CPE of moderate and high intensity (Grassi et al. 1996; Krustrup et al. 2009). This implies that oxygen delivery limitations do not play a significant role in these conditions. However, this does not need to be the case in the whole‐body (e.g., cycling) step‐incremental and ramp‐incremental exercise, especially at higher work intensities (Rossiter 2011).

It has been postulated that a major mechanism responsible for the regulation of the skeletal muscle and heart bioenergetic system, especially oxidative phosphorylation (OXPHOS), during work transitions is each‐step activation (ESA) (Korzeniewski 1998, 2003, 2007, 2017b; Korzeniewski and Zoladz 2004; Korzeniewski and Rossiter 2015). According to this mechanism, not only ATP usage and NADH supply but also all OXPHOS complexes (complex I, complex III, complex IV, ATP synthase, ATP/ADP carrier, and P_i_ carrier) and glycolysis are directly activated by some cytosolic mechanism during the rest‐to‐work or low‐to‐high work transition in skeletal and heart muscle cells. While ESA is essentially the only mechanism acting in intact heart in situ, where metabolite concentrations are approximately constant during work transitions, in skeletal muscle it cooperates with the negative feedback through the increase in ADP and P_i_ (Liguzinski and Korzeniewski 2006; Korzeniewski 2017b).

It was postulated that the ESA intensity (*A*_OX_) depends in a saturating manner on the ATP usage activity (*A*_UT_) during voluntary CPE in humans (Korzeniewski 2018). Such a saturating‐type *A*_OX_‐*A*_UT_ dependence, when supplemented with the additional ATP usage above CP, underlying the slow component of the $\dot{V}O_{2}$ on‐kinetics (Korzeniewski and Rossiter 2015; Korzeniewski and Zoladz 2015), was able to represent correctly various kinetic properties of the skeletal muscle bioenergetic system in humans during constant‐power voluntary exercise (Korzeniewski 2018). On the other hand, the power‐type *A*_OX_‐*A*_UT_ relationship seems to be present in electrically stimulated skeletal muscle (Korzeniewski 2018). It was postulated that the difference between these two systems is due to different muscle fibers recruitment patterns (Korzeniewski 2018).

The first aim of this theoretical study is to propose the mechanism(s) underlying the $\dot{V}O_{2}$‐PO nonlinearity in skeletal muscle and the differences in the size of this nonlinearity in various exercise modes. In particular, the following exercise modes are considered explicitly: constant‐power exercise (CPE) (300 W, lasting 6 min), step‐incremental exercise (SIE) (30 W/3 min), as well as slow (10 W/min), moderate (30 W/min), and fast (50 W/min) ramp‐incremental exercise (RIE). The second aim is to decide whether the regulation of OXPHOS is the same in CPE, on the one hand, and in SIE and RIE, on the other hand. Third, this study is intended to predict the changes over time of muscle $\dot{V}O_{2}$, ADP, PCr, P_i_, and pH in all considered exercise modes. These variables were not measured experimentally in SIE and RIE, mostly because of technical difficulties.

It was assumed, in order to reproduce appropriately experimental data, that while the saturating‐type *A*_OX_‐*A*_UT_ relationship is present in CPE, then the kinetic properties of the skeletal muscle bioenergetic system in SIE and RIE are better explained by the declining‐type *A*_OX_‐*A*_UT_ relationship. It was assumed that the muscle $\dot{V}O_{2}$‐PO nonlinearity and its magnitude is related to the increase in $\dot{V}O_{2}$ due to the time‐ and PO‐dependent increase in the additional ATP usage underlying the slow component of the $\dot{V}O_{2}$ on‐kinetics minus the decrease in $\dot{V}O_{2}$ due to an increase in ATP supply by anaerobic glycolysis.

It is hypothesized that for the assumptions made within the model the muscle the $\dot{V}O_{2}$‐PO nonlinearity will be greatest and positive (upward deviation) in CPE (300 W, 6 min), smaller in SIE (30 W/3 min), even smaller in slow RIE (10 W/min), in medium RIE (30 W/min), and smallest (in fact negative, downward deviation) in fast RIE (50 W/min). It is hypothesized that the *A*_OX_‐*A*_UT_ relationship is different in CPE, on the one hand, and in SIE and RIE, on the other hand, due to different muscle fibers recruitment patterns. It is expected that (end‐step) PCr will decrease and (end‐step) P_i_ will increase linearly with time and PO in SIE and in RIEs of different steepness below CP, while these changes will accelerate moderately above CP. It is expected that the increase in ADP and decrease in pH will significantly accelerate with PO and time at all PO values.

Generally, it is postulated that the size of the muscle $\dot{V}O_{2}$‐PO nonlinearity in various exercise modes is proportional to the intensity of the additional ATP usage minus the intensity of ATP supply by anaerobic glycolysis. It is proposed that the extent of the additional ATP usage is proportional to the time integral of the PO ‐ CP difference above CP. It is argued that the regulation of OXPHOS is different in CPE, on the one hand, and in SIE and RIE, on the other hand.

## Theoretical Methods

### Abbreviations

The following abbreviations are used throughout the text: *A*_OX_, ESA intensity (relative OXPHOS activity); *A*_UT_, relative ATP usage activity; CP, critical power; CPE, constant‐power exercise; ESA, each‐step activation; OXPHOS, oxidative phosphorylation; PO, power output; SIE, step‐incremental exercise; RIE, ramp‐incremental exercise; *τ*_p_, characteristic transition time of the primary phase of the $\dot{V}O_{2}$ on‐kinetics; and *t*_0.63_, time to reach 63% of the $\dot{V}O_{2}$ amplitude, analogous to *τ*_p_. Other abbreviations are used only in equations within this Theoretical Methods section and are defined in the place of their appearance.

### Ethical approval

This is a purely theoretical study that did not involve any experiments on humans or animals.

### Computer model

The computer model of OXPHOS and the entire bioenergetic system in intact skeletal muscle (Korzeniewski and Zoladz 2001; Korzeniewski and Liguzinski 2004; Korzeniewski and Rossiter 2015; Korzeniewski 2018) was used in the simulations carried out in this study. This model comprises explicitly the NADH supply block (TCA cycle, fatty‐acid *β*‐oxidation, MAS, etc.), particular OXPHOS complexes (complex I, complex III, complex IV, ATP synthase, ATP/ADP carrier, and P_i_ carrier), proton leak through the inner mitochondrial membrane, glycolysis (aerobic and anaerobic), ATP usage, creatine kinase (CK), and proton efflux/influx to/from blood. The complete model description is located on the website: http://awe.mol.uj.edu.pl/~benio/.

### Computer simulations

#### Each‐step activation (ESA) intensity‐ATP usage activity dependence

The relative activity of ATP usage *A*_UT_ (relative increase in its rate constant *k*_UT_ in relation to rest) between 1 (rest) and 105 (maximum *A*_UT_) was used in computer simulations.

The phenomenological (averaged over whole muscle) dependence of *A*_OX_ on *A*_UT_ is most probably different in various modes of muscle work and depends on the pattern of muscle fiber recruitment by motor units (Henneman et al. 1965a,b) at different work intensities. In electrically‐stimulated muscle during CPE all fiber types (predominantly oxidative type I and IIa fibers with high ESA intensity as well as predominantly glycolytic type IIx and IIb fibers with low ESA intensity) are recruited simultaneously at all stimulation frequencies/work intensities/ATP usage activities. The increase in work intensity is simply due to the increase in stimulation frequency. It has been demonstrated (Korzeniewski 2018) that the kinetic properties of the bioenergetic system in electrically stimulated muscle can be satisfactorily accounted for by the power‐type *A*_OX_‐*A*_UT_ relationship:

(1) $$A_{\mathrm{OX}}=A_{\mathrm{UT}}^{p_{\mathrm{OX}}}$$

where *A*_OX_ (ESA, each‐step activation intensity) is the relative OXPHOS (+ NADH supply) activity (activation in relation to rest), *A*_UT_ is the relative ATP usage activity (activation in relation to rest), and the power coefficient p_OX_ is a measure of ESA strength (*p*_OX_ = 0.45 was used in Korzeniewski 2018 and in this study).

In CPE during voluntary exercise (cortically stimulated muscle) a mixture of different fiber types is recruited at different work intensities, with the fraction of glycolytic type IIx and IIb fibers with low ESA intensity increasing with work intensity (power output). Thus, only or mostly oxidative type I and IIa fibers with high ESA intensity are recruited at the onset of exercise at low work intensities, while a mixture of oxidative type I and IIa fibers as well as glycolytic type IIx and IIb fibers with low ESA intensity is recruited at the onset of intense CPE in cortically stimulated muscle. Nevertheless, even at highest work intensities a mixture of oxidative and glycolytic fibers is recruited at the onset of exercise. It was shown (Korzeniewski 2018) that the kinetic system properties in this situation can be well reproduced using the saturating‐type *A*_OX_‐*A*_UT_ relationship:

(2) $$A_{\mathrm{OX}}=1+A_{\mathrm{OX}\;\;\max}\left(\frac{A_{\mathrm{UT}-1}}{\left(A_{\mathrm{UT}}-1\right)+K_{A_{\mathrm{UT}}}}\right)$$

where *A*_OX_ (ESA, each‐step activation intensity) is the relative OXPHOS (+ NADH supply) activity (direct activation in relation to rest), *A*_UT_ is the relative ATP usage activity (activation in relation to rest), *A*_OXmax_ = 5 is the maximum *A*_OX_−1 (thus, maximum *A*_OX_ = 6), and *K*_AUT_ = 5 is the half‐saturating *A*_UT_ value for the increase in *A*_OX_.

Finally, in SIE and RIE most probably only or mostly oxidative, fatigue‐resistant type I (and IIa) muscle fibers with high ESA intensity are recruited in the initial period of exercise at low power outputs below CP (Jones et al. 2010; Poole et al. 2016). As the exercise intensity increases with time above CP, still greater and greater proportion of glycolytic, fatigue‐susceptible type IIx and IIb muscle fibers with low ESA intensity is recruited. Finally, at highest power outputs, only or mostly type IIx and IIb muscle fibers are recruited. In order to express this system property within one‐compartment computer model used in this theoretical study, it was assumed that the overall phenomenological *A*_OX_‐*A*_UT_ dependence is rather declining than saturating in SIE and RIE. Such a declining‐type *A*_OX_‐*A*_UT_ relationship was represented in the form of the maximum ATP usage activity *A*_OXmax_ dependent on the relative ATP usage activity *A*_UT_:

(3) $$A_{\mathrm{OXmax}}=A_{\mathrm{OXmax}0}\left(\frac{A_{\mathrm{UT}0}-A_{\mathrm{UT}}}{A_{\mathrm{UT}0}}\right)$$

where *A*_OXmax0_ = 8 is the “initial” (for very low power outputs) *A*_OXmax_ and *A*_UT0_ = 206 is the “reference” ATP usage activity. The values of these parameters were adjusted in order to represent appropriately experimental data, for instance, the dependence of *τ*_p_ on *A*_UT_ (power output) in SIE and the size of the $\dot{V}O_{2}$‐PO nonlinearity in SIE and RIE, as well as reasonable values and their changes with time and PO of muscle $\dot{V}O_{2}$, ADP, PCr, P_i_, and pH in SIE and RIE.

**Figure 1:**
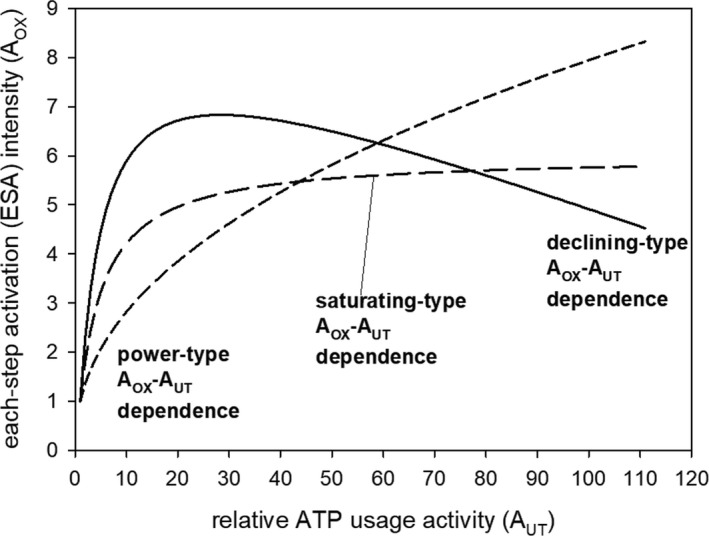
Three types of the *A*_OX_ (ESA, each‐step activation intensity)‐*A*_UT_ (relative ATP usage activity) relationship: power type, saturating type, and declining type. The power‐type *A*_OX_‐*A*_UT_ relationship was generated using Equation (1), saturating‐type *A*_OX_‐A_UT_ relationship using Equation (2), and declining‐type *A*_OX_‐*A*_UT_ relationship – using Equations (2) and (3).

**Figure 2:**
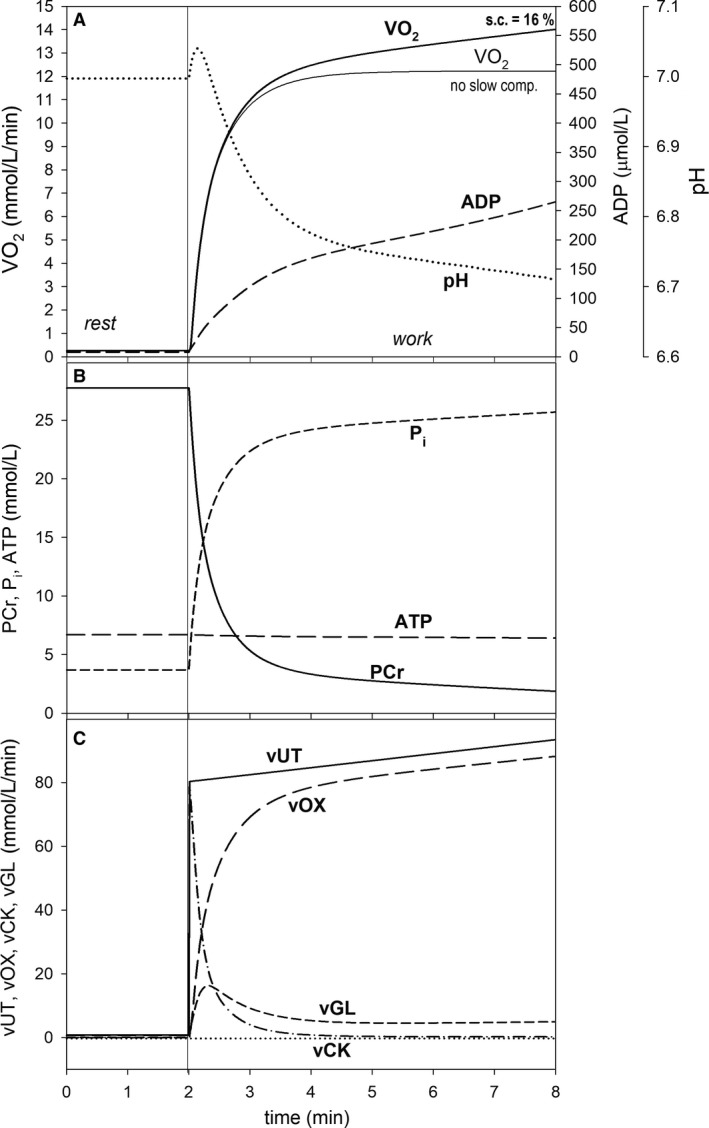
Simulated time courses of muscle bioenergetic system variables during rest‐to‐work transition for intense voluntary constant‐power exercise (CPE) (6 min) for standard parameter values. (A) Changes in muscle $\dot{V}O_{2}$ (in the presence, thick solid line, or absence, thin solid line, of the additional ATP usage and thus slow component of the $\dot{V}O_{2}$ on‐kinetics), cytosolic ADP, and pH; (B) changes in PCr, cytosolic ATP, and P_i_; (C) changes in total ATP usage (*v*UT) (normal ATP usage + additional ATP usage), ATP supply by OXPHOS (+ aerobic glycolysis) (vOX), ATP supply by anaerobic glycolysis (vGL), and ATP supply by creatine kinase (vCK). s.c., slow component of the $\dot{V}O_{2}$ on‐kinetics.

**Figure 3:**
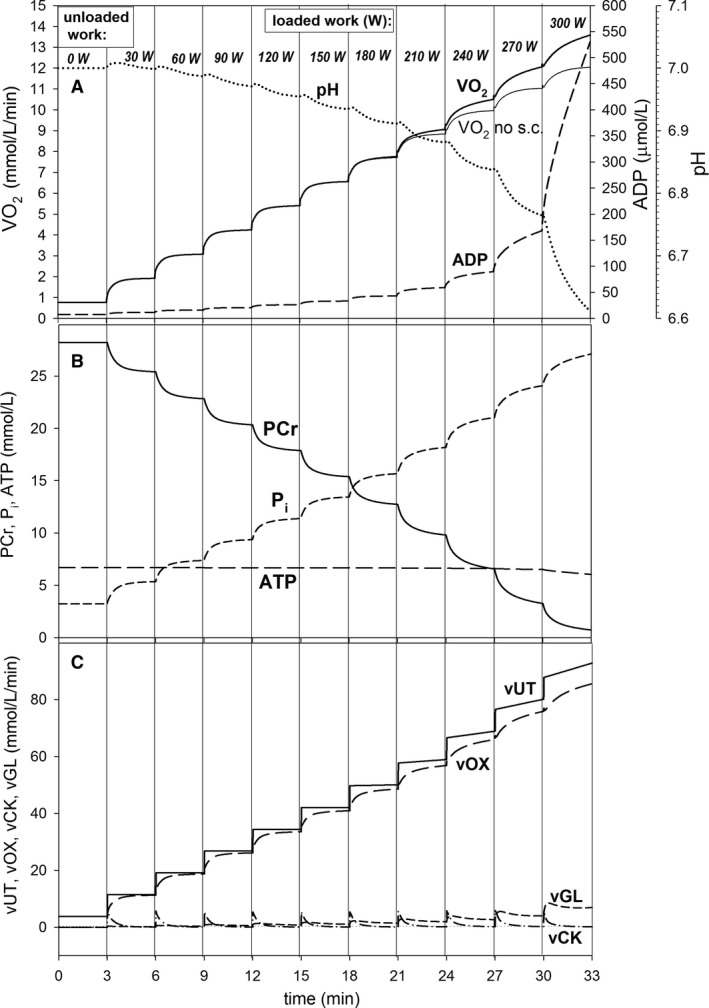
Simulated time courses of muscle bioenergetic system variables during step‐incremental exercise (SIE) (30 W/3 min) for standard parameter values. (A) Changes in muscle $\dot{V}O_{2}$ (in the presence, thick solid line, or absence, thin solid line, of the additional ATP usage and thus slow component of the $\dot{V}O_{2}$ on‐kinetics), cytosolic ADP, and pH; (B) changes in PCr, cytosolic ATP, and P_i_; (C) changes in total ATP usage (*v*UT), ATP supply by OXPHOS (+aerobic glycolysis), ATP supply by anaerobic glycolysis (vGL), and ATP supply by creatine kinase (vCK). s.c., slow component of the $\dot{V}O_{2}$ on‐kinetics.

**Figure 4:**
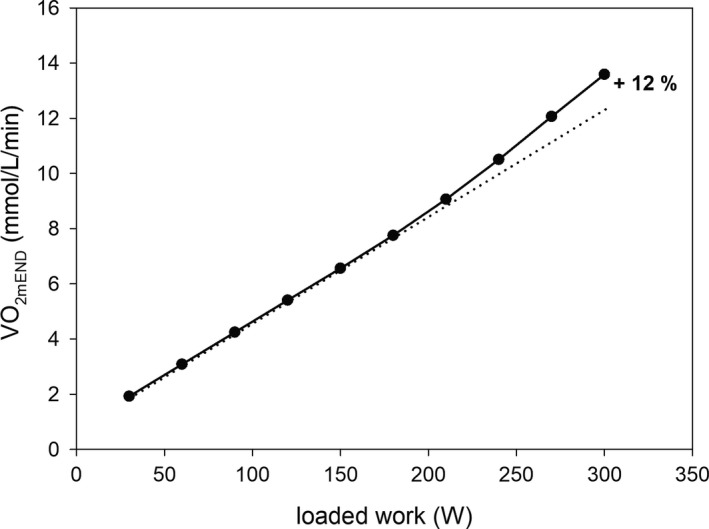
Simulated muscle $\dot{V}O_{2\mathrm{END}}$ ($\dot{V}O_{2}$ at the end of subsequent steps)‐power output (PO) relationship for step‐incremental exercise (SIE) extracted from Figure 3.

**Figure 5:**
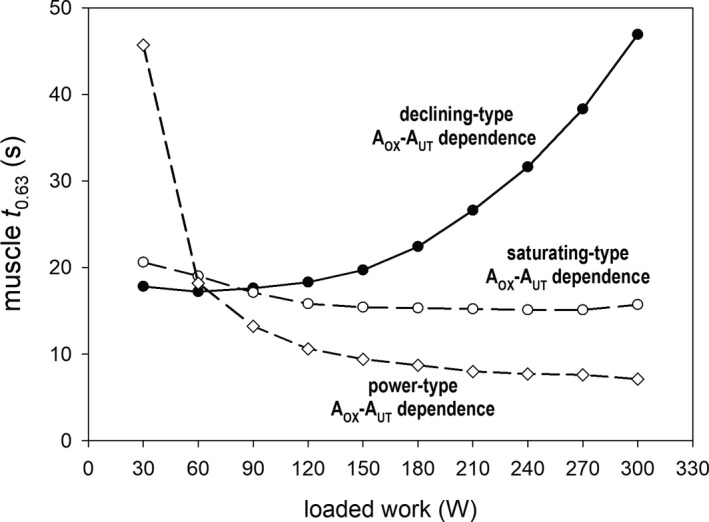
Simulated dependence of the characteristic transition time of the principal phase of the muscle $\dot{V}O_{2}$ on‐kinetics *t*_0.63_ (time to reach 63% of the $\dot{V}O_{2}$ amplitude, equivalent to *τ*_p_) on loaded work intensity (power output, PO) in step‐incremental exercise (SIE) in the absence of the additional ATP usage, and thus slow component of the $\dot{V}O_{2}$ on‐kinetics. Three types of the *A*_OX_ (ESA intensity)‐*A*_UT_ (ATP usage activity) relationships were used: power type (Eq. (1)), saturating type (Eq. (2)), and declining type (Eqs. (2) and (3)).

**Figure 6:**
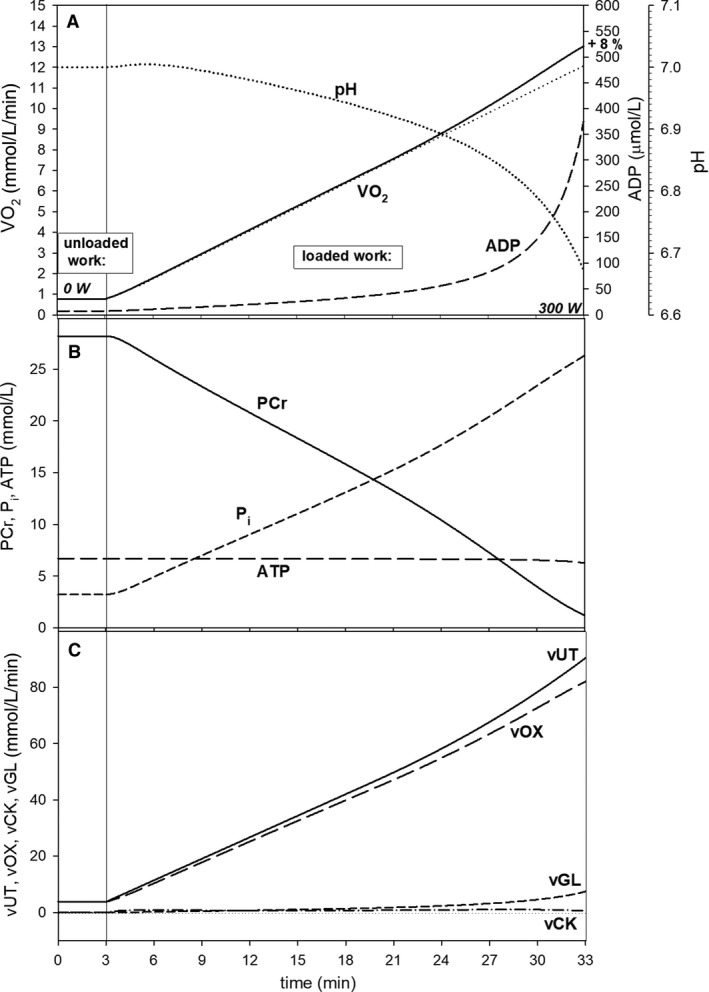
Simulated time courses of muscle bioenergetic system variables during slow ramp‐incremental exercise (RIE) (10 W/min) for standard parameter values. (A) Changes in muscle $\dot{V}O_{2}$, cytosolic ADP, and pH; (B) changes in PCr, cytosolic ATP, and P_i_; (C) changes in total ATP usage (*v*UT), ATP supply by OXPHOS (+ aerobic glycolysis), ATP supply by anaerobic glycolysis (vGL), and ATP supply by creatine kinase (vCK).

**Figure 7:**
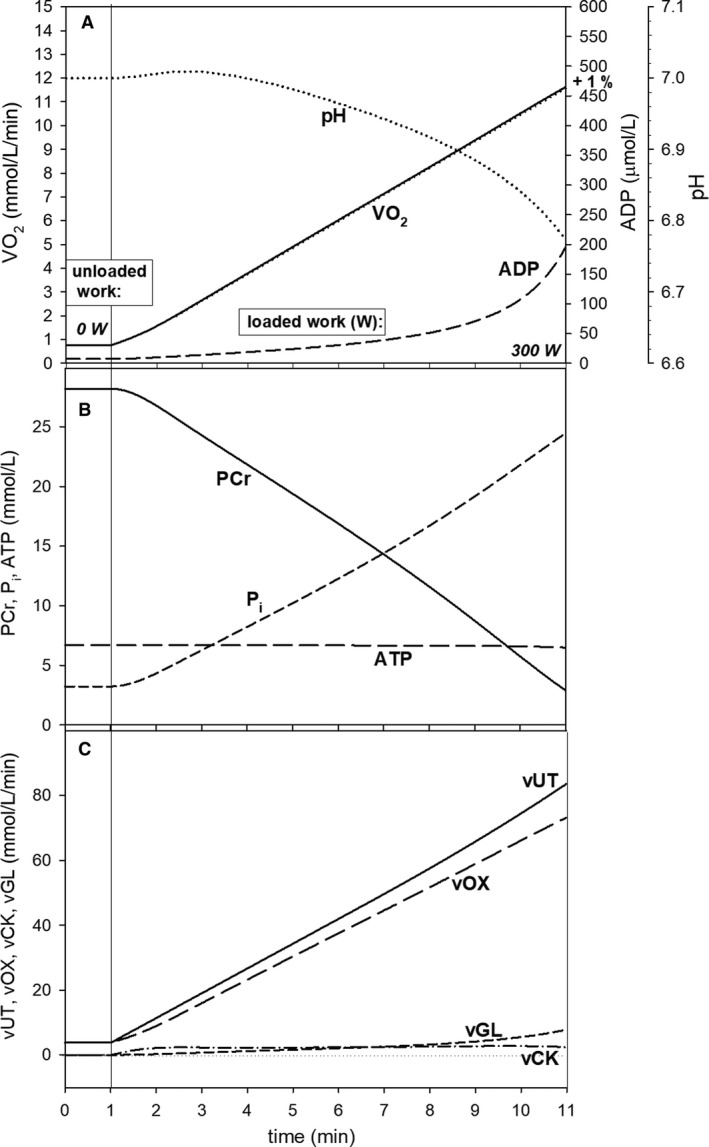
Simulated time courses of muscle bioenergetic system variables during medium ramp‐incremental exercise (RIE) (30 W/min) for standard parameter values. (A) Changes in muscle $\dot{V}O_{2}$, cytosolic ADP, and pH; (B) changes in PCr, cytosolic ATP, and P_i_; (C) changes in total ATP usage (*v*UT), ATP supply by OXPHOS (+aerobic glycolysis), ATP supply by anaerobic glycolysis (vGL), and ATP supply by creatine kinase (vCK).

**Figure 8:**
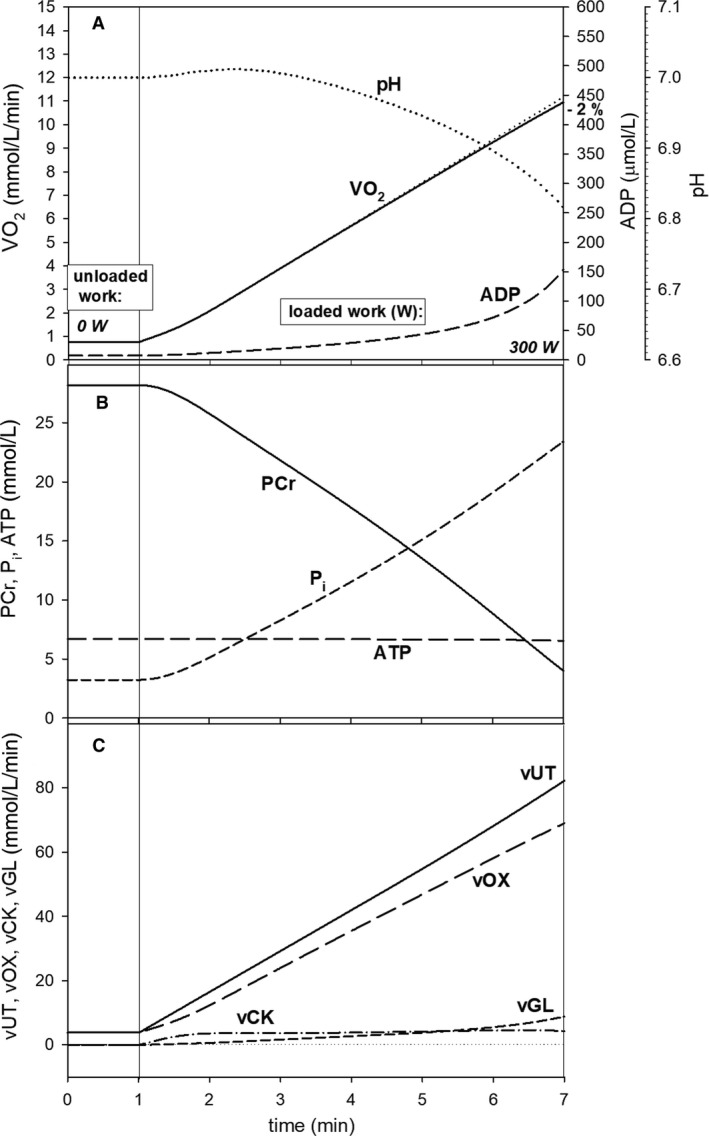
Simulated time courses of muscle bioenergetic system variables during fast ramp‐incremental exercise (RIE) (50 W/min) for standard parameter values. (A) Changes in muscle $\dot{V}O_{2}$, cytosolic ADP, and pH; (B) changes in PCr, cytosolic ATP, and P_i_; C, changes in total ATP usage (*v*UT), ATP supply by OXPHOS (+aerobic glycolysis), ATP supply by anaerobic glycolysis (vGL), and ATP supply by creatine kinase (vCK).

These three types of the *A*_OX_‐*A*_UT_ relationship are presented in Figure 1. In the simulation concerning voluntary CPE, presented in Figure 2, single values of *A*_OX_ and *A*_UT_, with *A*_OX_ calculated due to the saturating‐type *A*_OX_‐*A*_UT_ relationship (Eq. (2)) are used. In simulations concerning SIE and RIE, presented in Figures 3, 4, 5, 6, 7, 8, the declining type of the *A*_OX_‐*A*_UT_ relationship (Eqs. (2) and (3)) is used. In Figure 5, all three types were used for SIE for comparison.

#### ATP usage activity/power output

It was postulated that the additional ATP usage (Korzeniewski and Rossiter 2015), underlying the slow component of the $\dot{V}O_{2}$ on‐kinetics (Rossiter et al. 2002; Rossiter 2011; Korzeniewski and Rossiter 2015; Korzeniewski and Zoladz 2015), appears when the relative ATP usage activity A_UT_ exceeds the critical ATP usage activity (related to critical power; Jones et al. 2010; Poole et al. 2016). The relative (scaled to 1 at rest) *A*_UT_ between 1 (rest) and 105 (maximum *A*_UT_) was used in computer simulations. One *A*_UT_ unit is equivalent to ~3 W of power output (PO) for whole‐body exercise (e.g., cycling) (Korzeniewski 2018). It is assumed in this study that unloaded PO equals 12 W. Therefore, *A*_UT_ = 1 corresponds to rest (total PO = 0); *A*_UT_ = 5 (unloaded exercise) corresponds to total PO = 12 W, unloaded PO = 12 W, and loaded PO = 0 W; *A*_UT_ = 15 (first step in SIE) corresponds to total PO = 42 W, unloaded PO = 12 W, and loaded PO = 30 W; *A*_UT_ = 105 (maximum work) corresponds to total PO = 312 W, unloaded PO = 12 W, and loaded PO = 300 W.

The additional ATP usage, postulated to underlie in CPE and SIE the slow component of the $\dot{V}O_{2}$ on‐kinetics, appears when the relative ATP usage activity *A*_UT_ exceeds the critical relative ATP usage activity *A*_UTcrit_ (and thus power output exceeds critical power) (Korzeniewski and Rossiter 2015; Korzeniewski and Zoladz 2015; Korzeniewski 2018). The rate (*vv*_UTadd_) of the increase in the absolute additional ATP usage flux (rate in mmol/L min^−1^) *v*_UTadd_ is described in this study by the following equation:

(4) $$vv_{\mathrm{UTadd}}=k_{\mathrm{UTadd}}\cdot v_{\mathrm{UT}}\cdot \left(A_{\mathrm{UT}}-A_{\mathrm{UTcrit}}\right)$$

where *vv*_UTadd_ (mmol/L min^−2^) is the rate of the increase in the absolute additional ATP usage flux (in mmo/L min^−1^) (*v*_UTadd_), *k*_UTadd_ = 0.0005 min^−1^ is the “rate constant” of the increase in the absolute additional ATP usage flux in time, *A*_UT_ is the relative normal (without additional ATP usage) ATP usage activity (activation in relation to rest) (unitless) and *A*_UTcrit_ = 60 (unitless) is the critical relative ATP usage activity (corresponding to critical power, that is total PO = 177 W and loaded PO = 165 W). This is the kinetic description of the activity of the additional ATP usage used in Korzeniewski (2018) for whole‐body CPE, modified in order to take into account the increase of *A*_UT_ (and therefore PO) with time in SIE and RIE. This equation implies that the additional ATP usage flux *v*_UTadd_ increases both with time and with power output (ATP usage activity). In other words, the current additional ATP usage is proportional to the time integral of *A*_UT_ ‐ *A*_UTcrit_, or, what is equivalent, to the time integral of power output over critical power (the PO ‐ CP difference) above CP. The parameter values, especially *k*_UTadd_, were chosen in order to give realistic values of the $\dot{V}O_{2}$‐PO nonlinearity.

The total absolute ATP usage flux *v*_UTtot_ (in mmol/L min^−1^) is equal to the sum of the normal and additional absolute ATP usage flux:

(5) $$v_{\mathrm{UTtot}}=v_{\mathrm{UT}}+v_{\mathrm{UTadd}}$$

#### Work transitions in constant‐power, step‐incremental, and ramp‐incremental exercise

The transition from rest (*A*_UT_ = 1) to maximal work with the relative ATP usage activity *A*_UT_ = 105 (total PO = 312 W, loaded PO = 300 W) was simulated in CPE. This is demonstrated in Figure 2. The rate constant of ATP usage *k*_UT_ was increased *A*_UT_ times in relation to rest. At the same time the activity of OXPHOS and NADH supply was elevated *A*_OX_ times (the rate constants of complex I: *k*_C1_, complex III: *k*_C3_, complex IV: *k*_C4_, ATP synthase: *k*_SN_, ATP/ADP carrier: *k*_EX_, P_i_ carrier *k*_PI_, and NADH supply: *k*_DH_ were increased *A*_OX_ times) in relation to rest. Glycolysis activity was increased A_GL_ times (the rate constant of glycolysis *k*_GL_ was increased *A*_GL_ times) in relation to rest. *A*_OX_ = 5.77 was calculated from Equation (2), while *A*_GL_ equaled $A_{\mathrm{UT}}^{0.55}=12.93$ (Korzeniewski 2018).

An instantaneous increase in the activity of ATP usage (*A*_UT_‐fold increase in *k*_UT_) during on‐transient was applied in computer simulations for voluntary CPE. The additional ATP usage activity increased with time according to Equation (4). On the other hand, some delay in the increase in the activity (ESA) of OXPHOS and glycolysis during on‐transient was assumed (it should be stressed that the small delay in the increase in OXPHOS activity is not equivalent to the increase in $\dot{V}O_{2}$ related to both ESA and ADP and P_i_ increase). The time‐dependent activation after the onset of elevated work in CPE was described by the following equation (Korzeniewski and Rossiter 2015):

(6) $$m_{X}=A_{X}-\left(A_{X}-1\right)\cdot e^{-t/t{\left(\mathrm{ON}\right)}_{X}}$$

where *X* is OX (oxidative phosphorylation + NADH supply) or GL (glycolysis), *m*_*X*_ is the current (at time *t*) relative activity of *X* (multiplicity of the rest value(s) of its rate constant(s)), *t*(ON)_*X*_ is the characteristic activation time of *X*, and *t* is the time after the onset of exercise (rest‐to‐work transition), *t*(ON)_OX_ = 3 sec and t(ON)_GL_ = 6 sec (Korzeniewski and Rossiter 2015). The simulated CPE lasted 6 min. In order to determine the time to reach 63% of the $\dot{V}O_{2}$ amplitude *t*_0.63_ (analogous to *τ*_p_), a computer simulation without the additional ATP usage was carried out.

**Figure 9:**
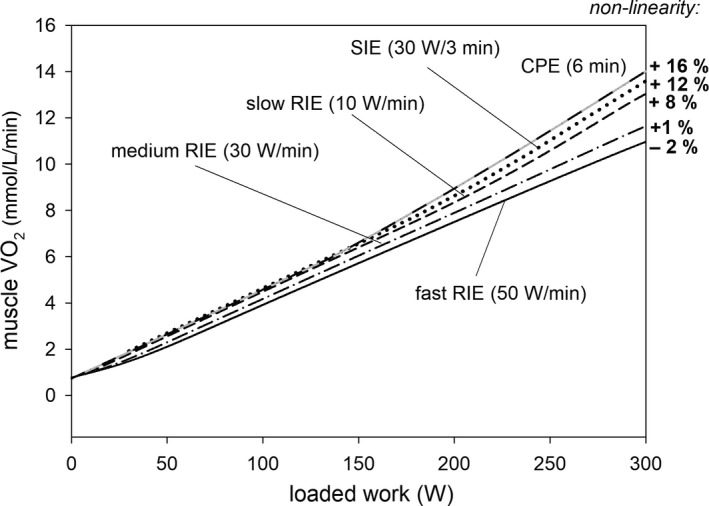
Simulated dependence of muscle $\dot{V}O_{2}$ (end‐exercise for CPE, end‐step for SIE, and during exercise for RIE) on loaded work (power output) for slow (10 W/min), medium (30 W/min), and fast (50 W/min) ramp‐incremental exercise (RIE) as well as for step‐incremental exercise (SIE) (30 W/3 min) and constant‐power exercise (CPE) (6 min).

Also a series of computer simulations for CPE, assuming the saturating‐type *A*_OX_‐*A*_UT_ relationship, was carried out in order to determine the muscle $\dot{V}O_{2}$‐PO dependence for CPE shown in Figure 9. In subsequent simulations *A*_UT_ was increased from 5 (0 W loaded PO, 12 W total PO) to 105 (300 W loaded PO, 312 W total PO). The simulated exercise lasted 6 min.

Step‐incremental exercise (Fig. 3) was started in computer simulations from the baseline representing unloaded work with *A*_UT_ = 5 (total PO = 12 W, loaded PO = 0 W, and unloaded PO = 12 W). *A*_UT_ was increased instantaneously by 10 *A*_UT_ units (30 W) after every 3 min. The activity of OXPHOS (and NADH supply) was increased in subsequent steps *A*_OX_ times in relation to rest, according to Equations (2) and (3) (using the declining‐type *A*_OX_‐*A*_UT_ relationship). The relative activity of glycolysis *A*_GL_ equaled *A*_UT_^0.55^. The time‐dependent activation of OXPHOS and glycolysis after the onset of elevated work in subsequent steps was described by the following equation:

(7) $$m_{X}=A_{X}^{n}-\left(A_{X}^{n}-A_{X}^{n-1}\right)\cdot e^{-t/t{\left(\mathrm{ON}\right)}_{X}}$$

where *X* is OX (oxidative phosphorylation + NADH supply) or GL (glycolysis), *m*_*X*_ is the current (at time *t*) relative activity of *X* (multiplicity of the rest value(s) of its rate constant(s)), *A*_*X*_^*n*^ is the *X* activity in a given step *n*,* A*_*X*_^*n*−1^ is the *X* activity in the previous step *n*−1, *t*(ON)_*X*_ is the characteristic activation time of *X*, and *t* is the time after the onset of elevated work in step *n*,* t*(ON)_OX_ = 3 sec and *t*(ON)_GL_ = 6 sec, as for CPE. The additional ATP usage activity increased above the critical ATP usage activity (critical power) according to Equation (4). In order to determine *t*_0.63_ for each step, a computer simulation without the additional ATP usage (and thus the slow component of the $\dot{V}O_{2}$ on‐kinetics) was made. However, this procedure gave only approximate estimations of *t*_0.63_ values for particular steps in the presence of the additional ATP usage shown in Figure 3.

All three types of the *A*_OX_‐*A*_UT_ relationships: power type (Eq. (1)), saturating type (Eq. (2)), and declining type (Eqs. (2) and (3)) were used for step‐incremental exercise in order to compare their effect on muscle *t*_0.63_‐PO relationship presented in Figure 5. No additional ATP usage, and therefore slow component of the $\dot{V}O_{2}$ on‐kinetics, was assumed in order to extract the “pure” principal phase of this kinetics.

Ramp‐incremental exercise was started from the baseline *A*_UT_ = 5 (total PO = 12 W, loaded PO = 0 W, and unloaded PO = 12 W) representing unloaded work. In three different protocols (slow RIE, medium RIE, and fast RIE) *A*_UT_ increased linearly with time by 3.33, 10, and 16.67 *A*_UT_ units (10, 30, and 50 W) per min, respectively (Figs. 6, 7, 8). The relative activity of OXPHOS (and NADH supply) *A*_OX_ increased according to Equations (2) and (3). The relative activity of glycolysis *A*_GL_ equaled $A_{\mathrm{UT}}^{0.55}$. The additional ATP usage rate above the critical ATP usage activity *A*_UTcrit_ (equivalent to critical power) increased in time according to Equation (4).

It must be stressed that the same set of parameter values was used in all computer simulations concerning constant‐power exercise, step‐incremental exercise, and ramp‐incremental exercise. Therefore, in real situation they could concern one particular human subject. For this reason, the results of simulations for different exercise modes can be directly compared.

The oxygen concentration O_2_ = 30 *μ*mol/L was assumed in all simulations.

## Theoretical Results

First, CPE for the standard parameter values and maximum loaded power output (300 W; *A*_UT_ = 105) used in this study was simulated in order to establish a reference frame for further simulations concerning step‐incremental exercise and ramp‐incremental exercise. The theoretical results are presented in Figure 2. After the initial large and quick increase in muscle $\dot{V}O_{2}$ (due to ESA and rise in ADP and P_i_), a clear slow component of the muscle $\dot{V}O_{2}$ on‐kinetics can be observed (Fig. 2A), which was due to the additional ATP usage that increased linearly with time (Fig. 2C). ADP and P_i_ rose significantly and rapidly immediately after the onset of exercise, and then increased with a lower pace (Fig. 2A and B). The elevated ADP and P_i_ further stimulated ATP supply by OXPHOS (and glycolysis) and thus allowed to match the continuously increasing ATP usage (the additional ATP usage). PCr and pH slowly decreased with time after the initial significant fall (pH transiently rose during first 20 sec of exercise due to proton uptake by the creatine kinase‐catalyzed reaction) (Fig. 2A and B). ATP was supplied mostly by creatine kinase (CK) at the onset of exercise, but ATP synthesis was quickly taken over by OXPHOS and aerobic glycolysis (Fig. 2C). Glycolysis (aerobic + anaerobic) was significantly directly activated at the onset of exercise (ESA of glycolysis), and afterwards was further activated by an increase in ADP (within the model the explicit dependence on ADP involves the implicit dependence on AMP, P_i_, and other factors), and finally started to be significantly inhibited by accumulating protons (a third‐order dependence on H^+^: see Korzeniewski and Rossiter 2015). As a result, ATP supply by anaerobic glycolysis first increased during first about 20 sec of exercise (reaching at the peak almost 20% of total ATP supply) and then started to decline significantly (Fig. 2C).

The simulated additional $\dot{V}O_{2}$ over the principal phase of the $\dot{V}O_{2}$ on‐kinetics (shown in Fig. 2A as a thin solid line) in CPE, that is the slow component of the $\dot{V}O_{2}$ on‐kinetics, reaches 16% after 6 min of exercise (Fig. 2A). Of course, if a quicker increase in the additional ATP usage with time and PO is assumed, that is a higher rate of the increase of the additional ATP usage flux is used, a more pronounced slow component of the $\dot{V}O_{2}$ on‐kinetics (and greater $\dot{V}O_{2}$‐PO nonlinearity) would appear. For the assumed high work intensity (*A*_UT_ = 105, loaded PO = 300 W) and the size of the additional ATP usage (Eq. (4)) the constant‐power exercise demonstrated in Figure 2 could not be lengthened to, for example, 30 min (like 30 W/3 min SIE or slow 10 W/min RIE), as much earlier a muscle fatigue would take place because of a huge rise of ADP and P_i_, and fall of PCr and pH or perhaps of other factors (1).

The simulated characteristic transition time of the principal phase of the muscle $\dot{V}O_{2}$ on‐kinetics *t*_0.63_ for transition from unloaded work (12 W), and thus zero loaded work (0 W), to 300 W of loaded work in CPE equaled ~ 24 sec for the parameter values used in this simulation.

In SIE the slow component of the muscle $\dot{V}O_{2}$ on‐kinetics appeared in the steps with PO above CP. This is demonstrated in Figure 3A. PO in subsequent steps increased by 30 W after each 3 min. The end‐step (at the end of particular steps) PCr decreased linearly with time and PO, while end‐step P_i_ increased linearly with time and PO below CP. These changes accelerated moderately above CP (Fig. 3B). On the other hand, the increase in the end‐step ADP and decrease in the end‐step pH accelerated significantly with time and PO at all POs, especially at highest PO values (Fig. 3A). The additional ATP usage appeared in the steps with PO > CP (Fig. 3C). This phenomenon, together with the declining‐type *A*_OX_‐*A*_UT_ relationship, underlay (and determined the magnitude of) the slow component of the muscle $\dot{V}O_{2}$ on‐kinetics and accelerated changes in PCr and P_i_ above CP. Generally, OXPHOS was the main ATP supplier in this type of exercise. CK supplied some ATP at the beginning of each step, while the contribution of anaerobic glycolysis to ATP production was generally rather small, although its absolute value increased with PO (Fig. 3C). Nevertheless, the declining‐type *A*_OX_‐A_UT_ relationship increased the contribution of anaerobic glycolysis to ATP supply and enlarged changes in metabolites and pH.

The slow component of the muscle $\dot{V}O_{2}$ on‐kinetics above CP leads to a significant nonlinearity of the muscle and pulmonary end‐step $\dot{V}O_{2}$‐PO relationship in SIE (for instance, 30 W/3 min) (see Zoladz et al. 1995). This is demonstrated in Figure 4, where the dependence of the end‐step $\dot{V}O_{2}$ on power output (PO) extracted from Figure 3 is presented. One can see that in this case this nonlinearity equals 12%. Of course, this nonlinearity would be greater if a faster increase in the additional ATP usage were assumed (not shown).

The simulated dependence of *t*_0.63_ of the $\dot{V}O_{2}$ on‐kinetics on PO is strongly nonlinear in SIE for the declining‐type *A*_OX_‐A_UT_ relationship. This is demonstrated in Figure 5. *t*_0.63_ increases from 18 to 48 sec (2.7‐fold) between 30 and 300 W of loaded work (power output). However, these values are only approximate, as they were obtained in a computer simulation for SIE without the additional ATP usage, and thus the slow component of the $\dot{V}O_{2}$ on‐kinetics. On the other hand, when the saturating‐type *A*_OX_‐*A*_UT_ relationship, postulated to be present in voluntary constant‐power exercise (Korzeniewski 2018), was used in SIE, *t*_0.63_ was little dependent on power output (PO): a slight decrease in its value with PO from about 20 to 15 sec can be observed. Finally, when the power‐type *A*_OX_‐*A*_UT_ relationship, postulated to be present in electrically stimulated muscle (Korzeniewski 2018), was applied, *t*_0.63_ dropped very significantly with PO from 47 to 7 sec (Fig. 5).

In slow (10 W/min) RIE the increase in muscle $\dot{V}O_{2}$ with time and PO deviated from linearity for PO > CP. This is demonstrated in Figure 6A. This nonlinearity reached 8% at loaded PO = 300 W and resulted from the additional ATP usage above CP proportional to the time integral of the PO−CP difference (Fig. 6C) as well as from the declining‐type *A*_OX_‐*A*_UT_ relationship that increased the contribution of anaerobic glycolysis to ATP supply. The $\dot{V}O_{2}$‐PO nonlinearity would be even greater if not the ATP supply by anaerobic glycolysis that somewhat decreased $\dot{V}O_{2}$ at higher POs. PCr decreased and P_i_ increased linearly with time and PO below CP and these changes moderately accelerated above CP (Fig. 6B). On the other hand, the increase in ADP and decrease in pH accelerated with time and PO at all POs (Fig. 6A). The ATP usage increase with time and PO accelerated significantly above CP in a decidedly nonlinear manner (Fig. 6C). This was caused by the time‐ and PO‐dependent additional ATP usage that elevated the phenomenological ATP/PO ratio and thus phenomenological $\dot{V}O_{2}$/PO ratio. ATP was supplied mostly by OXPHOS (+ aerobic glycolysis), with a certain contribution of ATP synthesis by anaerobic glycolysis, especially at higher POs, and negligible contribution of ATP production by CK (Fig. 6C).

The simulated medium (30 W/min) RIE was qualitatively similar to slow RIE (10 W/min), although differed from it quantitatively. This is demonstrated in Figure 7. Overall (to the end of exercise) changes in $\dot{V}O_{2}$, PCr, cytosolic P_i_, ADP, and pH (Fig. 7A and B) were smaller, than in the previous case. The nonlinearity of the $\dot{V}O_{2}$‐PO relationship was much smaller. Although the same initial (0 W, unloaded work) and end‐exercise (300 W) loaded PO was used for both kinds of RIE, the exercise duration was threefold shorter for medium RIE (10 min), than for slow RIE (30 min), as the increase in PO in time was threefold faster in the former than in the latter. Within the model, it was assumed that the size of the additional ATP usage is not only PO‐dependent but also time‐dependent (compare Eq. (4)). Therefore, the additional ATP usage had much less time to develop during medium RIE than during slow RIE. In other words, the time integral of the PO ‐ CP difference above CP was much smaller. As a consequence, the end‐exercise additional ATP usage was much smaller in medium RIE than in slow RIE (however, again, the nonlinearity of the $\dot{V}O_{2}$‐PO relationship would be greater in the absence of ATP supply by anaerobic glycolysis). This, in turn, led to a much smaller positive nonlinearity of the muscle $\dot{V}O_{2}$‐PO relationship in medium RIE (+ 1%) than in slow RIE (+ 8%) (compare Figs. 6A and 7A). Finally, this was also the reason of smaller overall changes in metabolite levels and pH in the former than in the latter.

The simulated fast (50 W/min) RIE differed quantitatively (although not qualitatively) from slow RIE even more than medium RIE. This is demonstrated in Figure 8. The end‐exercise additional ATP usage was significantly smaller than in medium RIE, and even much more smaller than in slow RIE, as it had much less time to develop (the time integral of the PO ‐ CP difference was smaller). It was even smaller than ATP supply by anaerobic glycolysis (see Fig. 8C). While the additional ATP usage elevated $\dot{V}O_{2}$, ATP supply by anaerobic glycolysis diminished $\dot{V}O_{2}$. As a result, the nonlinearity of the muscle $\dot{V}O_{2}$‐PO relationship in fast RIE was negative (deviated downwards) (−2%), as the decrease in muscle $\dot{V}O_{2}$ due to the increase in ATP supply by anaerobic glycolysis prevailed at high POs over the increase in muscle $\dot{V}O_{2}$ due to the additional ATP usage. For the same reason, also metabolites and pH changed less for the same PO values than in slow and medium RIE (Fig. 8).

The dependence of muscle $\dot{V}O_{2}$ on loaded work (power output) was different for the three kinds of RIE, for SIE and CPE. This is demonstrated in Fig. 9. Muscle $\dot{V}O_{2}$ for a given PO was highest in CPE (6 min), lower in SIE (30 W/3 min), slow RIE (10 W/min), medium RIE (30 W/min), and lowest in fast RIE (50 W/min). This was caused by the highest end‐exercise additional ATP usage in CPE and lowest end‐exercise additional ATP usage in fast RIE. On the other hand, the relative contribution of anaerobic glycolysis (and creatine kinase) to ATP supply that lowers $\dot{V}O_{2}$ and thus diminishes the $\dot{V}O_{2}$‐PO nonlinearity was comparable in all discussed exercise modes (compare Figs. 3 and 6, 7, 8). The difference between particular curves would be greater, if lower ESA intensity, resulting in a longer *t*_0.63_ (*τ*_p_) for CPE, was used (not shown).

## Discussion

The present theoretical study dealt with three main problems: 1. The form of the *A*_OX_ (ESA, each‐step activation intensity)‐*A*_UT_ (ATP usage activity) relationship in skeletal muscle in different exercise modes; 2. The mechanisms underlying the nonlinearity of the muscle $\dot{V}O_{2}$‐PO relationship and the differences in its magnitude in various exercise modes. The following exercise modes were considered: whole‐body constant‐power exercise (CPE) (6 min), step‐incremental exercise (SIE) (30 W/min), as well as slow (10 W/min), medium (30 W/min), and fast (50 W/min) ramp‐incremental exercise (RIE), all with the same maximum loaded PO = 300 W; and 3. Time courses of muscle $\dot{V}O_{2}$, metabolites (cytosolic ADP, PCr, P_i_, and ATP), and cytosolic pH in CPE, SIE, and three modes of RIE.

Within the computer model used it was assumed that the muscle $\dot{V}O_{2}$‐PO nonlinearity constitutes a manifestation of the additional ATP usage magnitude minus ATP supply by anaerobic glycolysis. The additional ATP usage increased linearly with time and PO. Its magnitude was a derivative of the time integral of the PO ‐ CP difference above CP. Computer simulations demonstrated that various exercise modes considered in this study can be ordered in the following descending sequence taking into account the size of the muscle $\dot{V}O_{2}$‐PO nonlinearity: CPE (+16%) > SIE (+12%) > slow RIE (+8%) > medium RIE (+1%) > fast RIE (−2%). This theoretical study shows that the time‐ and PO‐dependent increase in the additional ATP usage can, at least potentially, explain the muscle $\dot{V}O_{2}$‐PO nonlinearity and account for the differences in its magnitude between various exercise modes encountered in experimental studies (see below). The $\dot{V}O_{2}$‐PO nonlinearity was lessened, especially at higher POs, by an increase in ATP supply by anaerobic glycolysis.

It was demonstrated that the declining‐type *A*_OX_‐*A*_UT_ relationship applied for SIE and RIE leads to a significant increase in *t*_0.63_ with PO in SIE. On the other hand, the saturating‐type *A*_OX_‐*A*_UT_ relationship, showed previously to describe well the system properties in CPE during voluntary exercise, results in *t*_0.63_ that depends little on PO (in fact slightly decreases with PO in SIE, but not in CPE). Additionally, the declining‐type *A*_OX_‐*A*_UT_ relationship leads to greater changes in metabolites and pH, greater contribution of anaerobic glycolysis to ATP supply, and lower (or more negative) $\dot{V}O_{2}$‐PO nonlinearity in relation to the saturating‐type *A*_OX_‐*A*_UT_ relationship. These predictions agree well with experimental data (see below). Therefore, it is postulated that the phenomenological (averaged over the entire muscle) regulation of OXPHOS is different in CPE, on the one hand, and in SIE and RIE, on the other hand. This difference is postulated to result from different muscle fibers recruitment pattern.

In CPE above CP, the simulated $\dot{V}O_{2}$, ADP, and P_i_ rose, whereas PCr and pH fell quickly and significantly after the onset of exercise (pH after an initial overshoot), and then changed with a moderate pace. This prediction agrees well with numerous experimental data (see, e.g., ref. 34 for discussion). Computer simulations show that, for the assumptions made, (end step) PCr decreases, while (end step) P_i_ increases linearly with time and PO below CP in SIE and RIE, and that these changes accelerate moderately above CP. On the other hand, the increase in (end step) ADP and decrease in (end step) pH accelerate significantly with time and PO at all PO values in SIE and RIE. These variables have not been measured in experimental studies in SIE and RIE yet.

### *A*_OX_‐*A*_UT_ relationship versus muscle fibers recruitment pattern

The muscle fiber recruitment pattern is most probably different in CPE in electrically stimulated muscle, in CPE during voluntary exercise as well as in SIE and RIE during voluntary exercise, as described in Theoretical Methods section. Within the one‐compartment computer model used in this study these differences were expressed in the form of different *A*_OX_‐*A*_UT_ relationships: power‐type relationship in electrically stimulated muscle, saturating type during voluntary CPE, and declining type during SIE and RIE.

### Muscle $\dot{\text{V}}{\text{O}}_{2}$ kinetics in constant‐power exercise

The simulated muscle $\dot{V}O_{2}$, following a large and quick initial rise, increased continuously with a slower pace after the onset of CPE of high intensity (loaded PO = 300 W). This is the so‐called slow component of the $\dot{V}O_{2}$ on‐kinetics (Poole et al. 1991, 1994). This property of the system is presented in Figure 2. As it can be seen, the extent of the slow component equaled 16% after 6 min of exercise. While the additional ATP usage was assumed to increase linearly with time from the onset of exercise (Fig. 2C), the time course of the muscle $\dot{V}O_{2}$ was more “rounded” (Fig. 2A) than the time course of ATP usage. This is because muscle $\dot{V}O_{2}$ was not stimulated directly by the additional ATP usage, but only indirectly through the increase in ADP and P_i_ (Fig. 2A and B).

If a faster increase in the additional ATP usage in time at a given power output (PO) was assumed (Eq. (4)), say by 60%, the magnitude of the slow component after 6 min of CPE with loaded PO of 300 W increased from 16% to 23% (not shown). On the other hand, the slow component could be diminished by elevated ATP supply by anaerobic glycolysis (not shown).

### Muscle $\dot{\text{V}}{\text{O}}_{2}$ kinetics and muscle $\dot{\text{V}}{\text{O}}_{2}$‐PO relationship in step‐incremental exercise

In computer simulations of SIE, the slow component of the muscle $\dot{V}O_{2}$ on‐kinetics appears above PO value (corresponding to CP), at which the additional ATP usage appears. This is demonstrated in Figure 3. A similar slow component is observed in experimental studies concerning cycling SIE (Keir et al. 2016). This property of the system leads to a nonlinearity in the end‐step $\dot{V}O_{2}$‐PO relationship. Figure 4 demonstrates the simulated nonlinearity in the end‐step muscle $\dot{V}O_{2}$‐PO relationship extracted from Figure 3. The extent of this nonlinearity at the end of exercise (at highest PO) equals 12%. A very similar nonlinearity in an experimental end‐step pulmonary $\dot{V}O_{2}$‐PO relationship was extracted in Korzeniewski (2018; Figure 9 therein) from Keir et al. (2016; Table 1 therein). Its extent equals 14%, which is similar to the simulated value. Such a phenomenon was reported previously for SIE (30 W/3 min) by Zoladz et al. (1995, 1998).

In the computer model used, the discussed nonlinearity in the muscle $\dot{V}O_{2}$‐PO relationship was mostly due to the additional ATP usage. The additional muscle $\dot{V}O_{2}$ related to this nonlinearity is somewhat diminished due to the increase in ATP supply by anaerobic glycolysis (and creatine kinase), especially at highest power outputs.

The simulated muscle $\dot{V}O_{2}$ on‐kinetics significantly slowed down with increasing PO in subsequent steps of SIE (30 W/3 min) for the declining type of the *A*_OX_‐*A*_UT_ relationship. This is demonstrated in Figure 5 that presents the dependence of *t*_0.63_ extracted from simulations concerning SIE without any additional ATP usage (and therefore slow component of the muscle $\dot{V}O_{2}$ on‐kinetics) for three types of the *A*_OX_‐*A*_UT_ relationship. For the declining type of this relationship, postulated to be relevant for SIE (and RIE), the muscle *t*_0.63_ increased 2.7‐fold from 18 sec at 30 W to 48 sec at 300 W. A noticeably greater relative lengthening of the transition time (*τ*_p_) of the primary phase of the pulmonary $\dot{V}O_{2}$ on‐kinetics from 21 to 98 sec (4.7‐fold increase) was encountered in Keir et al. (2016; Table 1 therein). However, the general muscle or pulmonary *t*_0.63_ (or *τ*_p_)‐PO dependence was similar both in computer simulations and experimental studies. On the other hand, *t*_0.63_ (*τ*_p_) slightly decreased with PO (from 20 to 15 sec) when the saturating‐type *A*_OX_‐*A*_UT_ relationship, postulated to be present in voluntary CPE, was used in the simulation concerning SIE (Fig. 5). The little PO‐dependent *t*_0.63_ (*τ*_p_) was encountered both in computer simulations (Korzeniewski 2018) and experimental studies (Poole and Jones 2005) concerning voluntary (cortical stimulation) CPE, although *t*_0.63_ slightly increased with PO in this type of exercise (for discussion see Korzeniewski 2018). Therefore, it seems that the regulation of OXPHOS, and in particular the phenomenological *A*_OX_‐*A*_UT_ relationship, is different in CPE, than in SIE (and, consequently, RIE). *t*_0.63_ decreased significantly with PO, when the power‐type *A*_OX_‐*A*_UT_ relationship, postulated to be present in electrically stimulated muscle, was applied (Fig. 5). Therefore, it seems evident that the declining‐type *A*_OX_‐*A*_UT_ relationship is most appropriate to account for the kinetic properties of the skeletal muscle bioenergetic system in SIE.

The first reason of the moderate quantitative difference between the relative increase with PO of the simulated muscle and experimental pulmonary *t*_0.63_ (*τ*_p_) can be of course the fact that the model used is (over)simplified, at least in the discussed aspect. This is a one‐compartment model (although it distinguishes the cytosol and mitochondrial compartments) that deals with variable (fluxes and metabolite concentrations) values averaged over entire muscle and all its fiber types. It has been postulated (Brittain et al. 2001) that in exercises starting from elevated work intensities a progressively greater fraction of glycolytic type II (especially type IIx and IIb) muscle fibers, characterized by a slow $\dot{V}O_{2}$ on‐kinetics (long *τ*_p_) (Jones et al. 2005), is recruited at the onset of exercise, which leads to lengthening of the overall muscle *τ*_p_. This explanation can be equally well applied to SIE. The model used in this study represents this phenomenon by using the declining type of the *A*_OX_‐*A*_UT_ dependence for SIE and RIE, instead of the saturating‐type *A*_OX_‐*A*_UT_ dependence postulated to be present in voluntary CPE (Korzeniewski 2018). Nevertheless, the representation of this property within the model is likely to be somewhat oversimplified. Second, the values of *t*_0.63_ for subsequent steps were estimated from a simulation that involved no additional ATP usage, and therefore no slow component of the $\dot{V}O_{2}$ on‐kinetics (thin line in Fig. 3A). This is likely to lead to an underestimation of *t*_0.63_ in steps with highest power outputs.

However, the difference between the relative increases in pulmonary and muscle *t*_0.63_ can be equally well real. A possible reason for the slower pulmonary than muscle $\dot{V}O_{2}$ on‐kinetics at high power outputs is a certain dissociation of the pulmonary and muscle $\dot{V}O_{2}$ on‐kinetics at high work intensities caused by limitations of O_2_ delivery by the circulation system and buffering by tissue, blood, and lung oxygen stores (Rossiter 2011; Keir et al. 2016). Additionally, the oxygen consumption by auxiliary tissues, such as heart, respiratory muscles, and posture‐maintaining muscles, can contribute significantly to the pulmonary $\dot{V}O_{2}$ kinetics, especially at high power outputs. Therefore, all three factors: the muscle fiber recruitment pattern, delays in oxygen supply by blood (together with buffering by tissue oxygen stores), and oxygen consumption by auxiliary tissues can contribute to the significant slowing down of the pulmonary $\dot{V}O_{2}$ on‐kinetics between low and high power output in SIE.

Taking into account all these reservations, a good, at least semiquantitative agreement can be observed between computer simulations (Fig. 5) and experimental data (Keir et al. 2016) concerning *τ*_p_ (*t*_0.63_) of the muscle and pulmonary $\dot{V}O_{2}$ on‐kinetics in subsequent steps in SIE.

Simulated *t*_0.63_ equaled ~ 24 sec for CPE for 0 → 300 W loaded work transition, assuming the saturating type of the *A*_OX_‐*A*_UT_ relationship. Therefore, *t*_0.63_ for transition to 300 W in CPE was decidedly lower, than *t*_0.63_ for transition to 300 W in step‐incremental exercise (SIE) for the declining type of the *A*_OX_‐*A*_UT_ relationship (48 sec). This finding further supports the conclusion that, unlike in CPE, the declining‐type, and not saturating‐type *A*_OX_‐*A*_UT_ relationship better accounts for the kinetic system properties in SIE.

### Muscle $\dot{\text{V}}{\text{O}}_{2}$ kinetics and muscle $\dot{\text{V}}{\text{O}}_{2}$‐PO relationship in ramp‐incremental exercise

It should be emphasized that in the case of RIE, when speaking about linearity or nonlinearity of the $\dot{V}O_{2}$‐PO dependence, the initial nonlinearity at the beginning of exercise is ignored. In other words, the problem concerns the “time‐shifted” $\dot{V}O_{2}$‐PO relationship (Keir et al. 2018).

The nonlinearity in the simulated muscle $\dot{V}O_{2}$‐PO relationship appeared also in RIE. Its extent was moderately positive (upward deviation) (but lower than in SIE) in slow RIE (8%) (Fig. 6), close to zero in medium RIE (1%) (Fig. 7), and slightly negative (downward deviation) in fast RIE (−2%) (Fig. 8). The extent of this nonlinearity, that is the value of the additional $\dot{V}O_{2}$, was determined by the additional ATP usage, proportional to the time integral of the PO ‐ CP difference, and diminished by the ATP supply by anaerobic glycolysis (and creatine kinase). As the latter effect prevailed over the former effect in fast RIE, a negative nonlinearity in the muscle $\dot{V}O_{2}$‐PO relationship was predicted in this exercise mode.

Generally, a similar, with a slight tendency to be more negative in fast RIE, nonlinearity in the experimental pulmonary $\dot{V}O_{2}$‐PO relationship was observed in different RIE modes. Keir et al. (2016) (Fig. 4 therein) encountered either approximately linear, slightly positively nonlinear or slightly negatively nonlinear pulmonary $\dot{V}O_{2}$‐PO relationship in different subjects for medium RIE (30 W/min). Takaishi et al. (1992) encountered a positive (+ 7%) pulmonary $\dot{V}O_{2}$‐PO nonlinearity for slow (10 W/min) RIE, approximately zero (0%) nonlinearity for medium‐slow (20 W/min) RIE, negative (−5%) nonlinearity for medium (30 W/min) RIE, and negative (−6%) nonlinearity for medium‐fast (40 W/min) RIE. Even higher positive (+ 11%) pulmonary $\dot{V}O_{2}$‐PO nonlinearity was encountered by Keir et al. (2018) for very slow (8 W/min) RIE, while the pulmonary $\dot{V}O_{2}$‐PO was essentially linear for medium RIE (30 W/min). Scheuermann et al. (2002) observed a high positive (+ 12%) pulmonary $\dot{V}O_{2}$‐PO nonlinearity for very slow (8 W/min) RIE and a negative (−5%) pulmonary $\dot{V}O_{2}$‐PO nonlinearity for very fast RIE (64 W/min). Davies et al. (1982) observed a small positive pulmonary $\dot{V}O_{2}$‐PO nonlinearity for medium‐slow (20 W/min) RIE and medium (30 W/min) RIE, and a small negative nonlinearity for fast (50 W/min) RIE (see Fig. 2 therein). Hansen et al. (1987) encountered a negative pulmonary $\dot{V}O_{2}$‐PO nonlinearity in a normal, albeit old subject (Fig. 3 therein). Whipp et al. (1981) observed either linear or slightly deviated downward (negative nonlinearity) pulmonary $\dot{V}O_{2}$‐PO relationship in fast RIE. Murgatroyd et al. (2014) encountered an approximately linear pulmonary $\dot{V}O_{2}$‐PO relationship in medium‐slow (20 W/min) RIE. Generally, the simulated muscle $\dot{V}O_{2}$‐PO relationships seem to agree rather well with experimental pulmonary $\dot{V}O_{2}$‐PO relationships. The experimental pulmonary $\dot{V}O_{2}$‐PO relationship in RIE tends to be positively nonlinear (upward deviation from linearity) in slow RIE, approximately linear in medium RIE and negatively nonlinear (downward deviation from linearity) in fast RIE. Perhaps the main slight difference between computer simulations and experimental data is that the negative nonlinearity in fast RIE seems frequently to be somewhat greater for experimental pulmonary $\dot{V}O_{2}$‐PO relationship than for simulated muscle $\dot{V}O_{2}$‐PO relationship (Fig. 8).

Again, the reason of this slight discrepancy between the simulated muscle and experimental pulmonary $\dot{V}O_{2}$ kinetics can be the fact that the computer model used for simulations is simplified in relation to the real system. Or, alternatively, the difference between the muscle and pulmonary $\dot{V}O_{2}$ kinetics can be real and result from limitations and delays in O_2_ transport by blood from working muscles to lungs and/or from replenishment of O_2_ stores in blood, tissues, and lungs (see, e.g., Rossiter 2011; Keir et al. 2016; Davies et al. 2017). The latter possibility can be supported by the finding that the Δ$\dot{V}O_{2}$/ΔPO slope was steeper in the second RIE episode than in the first RIE episode, separated by 10 min of cycling at 20 W (Jones and Carter 2004). This fact could suggest that the prior exercise stimulated blood circulation and O_2_ transport in the principal exercise, due to, for example, widening of blood vessels.

Overall, in the computer simulations the nonlinearity in the muscle $\dot{V}O_{2}$‐time and $\dot{V}O_{2}$‐PO relationship results mostly from the additional ATP usage above CP, while the extent of this nonlinearity: from the extent of the additional ATP usage minus the extent of ATP supply by anaerobic glycolysis. The additional ATP usage increasing linearly with time was postulated to underlie the slow component of the $\dot{V}O_{2}$ on‐kinetics in CPE (Rossiter 2011; Korzeniewski and Rossiter 2015; Korzeniewski and Zoladz 2015). In this study, the additional ATP usage that is both time‐ and PO‐dependent is postulated and therefore its size is proportional to the time integral of the PO ‐ CP difference above CP. The nonlinearity in the muscle $\dot{V}O_{2}$‐PO relationship is lessened by ATP supply by anaerobic glycolysis, especially at high POs. It is unlikely that, for example, elevated proton leak contributes significantly to the genesis and size of the muscle $\dot{V}O_{2}$ slow component and the muscle $\dot{V}O_{2}$‐PO nonlinearity, as the contribution of proton leak to muscle $\dot{V}O_{2}$ seems to be very small in skeletal muscle during intense exercise, although its contribution at rest is huge (Korzeniewski 2017a).

### Muscle $\dot{\text{V}}{\text{O}}_{2}$‐PO relationship in constant‐power, step‐, and ramp‐incremental exercise

CPE, SIE, and particular modes of RIE are characterized by different muscle $\dot{V}O_{2}$‐PO dependencies, as it can be seen in Figure 9. Muscle $\dot{V}O_{2}$ for a given PO was highest in CPE, lower in SIE, still lower in slow RIE, in medium RIE, and lowest in fast RIE. These differences would be even greater, if lower ESA intensity, resulting in a longer *t*_0.63_ (*τ*_p_) were assumed (not shown). The discussed phenomenon was due to the highest additional ATP usage at a given PO in CPE and lowest in fast RIE, especially at higher POs.

Qualitatively similar $\dot{V}O_{2}$‐PO relationships for different RIE modes were generated using a simple model by Keir et al. (2016) for pulmonary $\dot{V}O_{2}$ (Fig. 5B therein), although the differences between particular RIE modes were greater there. The authors explained these differences by time‐dependent recruitment of kinetically slower and less oxidatively efficient muscle fiber populations. Therefore, this explanation is partly different from the explanation proposed in this study based mostly on the time‐ and PO‐dependent increase in the additional ATP usage. On the other fact, both explanations involve the ATP supply by anaerobic glycolysis, the rise in which is related to fall in $\dot{V}O_{2}$. In fact, within the model the declining‐type *A*_OX_‐*A*_UT_ represents the recruitment of the glycolytic muscle fibers with low ESA intensity, and thus long *τ*_p_ (Korzeniewski 2003; Korzeniewski and Zoladz 2004), with high additional ATP usage increasing with time and PO, as well as with a high ATP supply by anaerobic glycolysis. The differences in the pulmonary $\dot{V}O_{2}$‐PO dependence between slow (10 W/min), medium‐slow (20 W/min), medium (30 W/min), and medium‐fast (40 W/min) RIE are rather small in experimental studies by Takaishi et al. (1992) (see Fig. 2 therein; notice that particular dependences are shifted vertically in order to avoid their overlapping), which resembles much more the theoretical results obtained in this study, than in Keir et al. (2016). Perhaps both mechanisms contribute to the discussed phenomenon.

It should be stressed that the muscle $\dot{V}O_{2}$‐PO relationship in SIE and RIE becomes more positively nonlinear (more deviated upward), when the saturating‐type *A*_OX_‐*A*_UT_ dependence, instead of the declining‐type *A*_OX_‐*A*_UT_ dependence, is used in computer simulations (not shown). The nonlinearity in this case is also slightly positive for fast RIE. As such kinetic behavior of the system differs more from the experimental pulmonary $\dot{V}O_{2}$‐PO relationship than the behavior generated using the declining‐type *A*_OX_‐*A*_UT_ dependence used in this study, it seems likely that also in RIE (like in SIE) the muscle $\dot{V}O_{2}$‐PO relationship is better accounted for by the declining‐type *A*_OX_‐*A*_UT_ dependence, than by saturating‐type *A*_OX_‐*A*_UT_ dependence. Therefore, the regulation of OXPHOS seems different in RIE and SIE, than in CPE, as discussed above.

### $\dot{\text{V}}{\text{O}}_{2\max}$ and PO_peak_ in different exercise modes

This study does not deal explicitly with the maximum oxygen consumption ($\dot{V}O_{2\max}$) and the power output achieved at it (PO_peak_), related to muscle fatigue, in CPE, SIE, and three modes of RIE. While the pulmonary $\dot{V}O_{2\max}$ seems to be identical in various exercise modes (medium RIE, SIE, and CPE), then PO_peak_ can differ very significantly, being highest in medium ramp‐incremental exercise (RIE) and lowest in constant‐power exercise (CPE) (8 min) for the same individual (Keir et al. 2018; Fig. 1 therein). The results of this theoretical study agree well with these experimental findings. This can be seen in Figure 9, where the simulated muscle $\dot{V}O_{2}$‐PO relationships for various exercise modes are presented. If it is assumed arbitrarily that muscle $\dot{V}O_{2\max}$ equals about 11.6 mmol/L min^−1^, then PO_peak_ would equal 300 W for medium RIE, 262 W for SIE, and 253 W for CPE. These differences would be much greater, if lower ESA intensity (resulting in a longer *t*_0.63_ for CPE), lower CP, and/or faster increase in the additional ATP usage with time and PO were used (not shown).

### Time courses of cytosolic metabolites and pH

While the simulated time courses of ADP, PCr, P_i_, and pH in CPE agree well with experimental data (see, e.g., ref. 34 for discussion), the simulated changes in metabolites and pH in SIE and RIE cannot be compared with experimental data, as these variables were not measured so far in SIE and RIE. Therefore, these model predictions will have to be verified by future experimental studies.

### Each‐step activation

An overall mechanism of parallel activation of ATP usage and ATP supply system in general during skeletal muscle contraction was first postulated by Hochachka (Hochachka and Matheson 1992; Hochachka 1994). It is supported by the observation (Chung et al. 2005) that the first phase of the $\dot{V}O_{2}$ increase after the onset of exercise is ADP independent, and therefore $\dot{V}O_{2}$ must be quickly elevated by some other mechanism. This mechanism was also confirmed by Wüst and co‐workers on the basis of the $\dot{V}O_{2}$‐ADP relationship during on‐transient in skeletal muscle (Wüst et al. 2011). Behnke et al. (2002) encountered a significant increase in $\dot{V}O_{2}$ immediately after the onset of electrical muscle stimulations. This agrees well with the ESA idea that predicts a very quick initial rise in muscle $\dot{V}O_{2}$ followed by an exponential increase due to the increase in ADP and P_i_ (Korzeniewski and Zoladz 2006).

Fell and Thomas (1995) proposed, in a more general and abstract way, an idea similar to ESA, called “multi‐site modulation”, in relation to other metabolic pathways, especially glycolysis and tricarboxilic acid (TCA) cycle. Therefore, it is likely that also particular enzymes within these metabolic pathways are directly activated during work transitions.

The each‐step activation (ESA) mechanism, being a special case of the parallel‐activation mechanism, was proposed in relation to OXPHOS in skeletal muscle by Korzeniewski (1998). According to this mechanism, not only ATP usage but also all OXPHOS complexes (complex I, complex III, complex IV, ATP synthase, ATP/ADP carrier, and P_i_ carrier), NADH supply block, and glycolysis/glycogenolysis are directly activated by some cytosolic mechanism, together with the stimulation of muscle contraction (actomyosin‐ATPase and Ca^2+^‐ATPase) by Ca^2+^ ions. The ESA mechanism is able to account for various, apparently not related to each other kinetic properties of the skeletal muscle and heart bioenergetic system, in particular OXPHOS (see Korzeniewski 2017b for review).

The molecular mechanism of ESA is still not fully known. Activation by matrix Ca^2+^ of three irreversible TCA (tricarboxylic acid) cycle dehydrogenases (pyruvate dehydrogenase, isocitrate dehydrogenase, and 2‐oxoglutarate dehydrogenase) (Hansford 1980; Denton and McCormack 1990) and by cytosolic Ca^2+^ of aralar, an element of the malate/aspartate shuttle (MAS) (Gellerich et al. 2012), has been demonstrated. Glancy et al. (2013) showed that Ca^2+^ elevates about twofold the activity of essentially all OXPHOS complexes in isolated skeletal muscle mitochondria incubated with glutamate/malate. However, it is not certain whether such an activation is sufficient to account for experimental data. A recent theoretical study (Korzeniewski and Rossiter 2015) suggests that OXPHOS complexes are activated directly over fivefold during rest‐to‐work transitions in humans. In other muscles or experimental conditions this direct activation of OXPHOS seems to be even much higher (Korzeniewski 2017b).

### Study limitations

Any computer model of a complex system can be at best only an approximation and simplification of the reality. As discussed above, the computer model used in this theoretical studies is a one‐compartment model that describes fluxes (e.g., oxygen consumption) and metabolite concentrations averaged over entire muscle and different muscle fiber types (type I, IIa, IIx, and IIb as well as their subtypes) with different patterns of recruitment in various modes of exercise (although it contains the cytosol and mitochondria compartment). On the other hand, the model was tested mostly for one‐compartment experimental data, like measured muscle $\dot{V}O_{2}$, PCr, cytosolic P_i_, ATP, and pH or calculated cytosolic ADP_free_. These variables are also averaged over various muscle fiber types.

A two‐compartment model of skeletal muscle bioenergetics was developed (Li et al. 2012), but it was also tested for one‐compartment data, and therefore could add little to one‐compartment models, being at the same time much more complicated and therefore much more difficult to verify.

In this work, the simulations for various exercise modes (CPE, SIE, and three rates of RIE) were made for the same parameter set and this is a great advantage that allows a direct comparison of the $\dot{V}O_{2}$‐PO nonlinearity and other kinetic properties. The theoretical results were also validated by comparison with various experimental data coming from different laboratories. This is another advantage, because the risk that some untypical set of experimental data was chosen as a reference frame is reduced.

Of course, a computer model cannot prove that the postulated mechanism underlying the $\dot{V}O_{2}$‐PO nonlinearity and the differences in its magnitude in various exercise modes, namely, the linear increase in the additional ATP usage with time and PO, is correct. Nevertheless, the model can demonstrate that this mechanism offers a plausible explanation that works at least semiquantitatively.

### Conclusions

It was postulated in this theoretical study that the assumed linear increase in the additional ATP usage with time and power output (PO) above critical power (CP) is the mechanism responsible for the nonlinearity of the muscle $\dot{V}O_{2}$‐PO relationship. Additionally, different extents of the additional ATP usage are proposed to be responsible for different magnitudes of this nonlinearity in different exercise modes. The size of the additional ATP usage was proportional to the time integral of the PO ‐ CP difference above CP. The extent of the muscle $\dot{V}O_{2}$‐PO nonlinearity was diminished by an increase in ATP supply by anaerobic glycolysis. Using the same parameter values, computer simulations predicted that the muscle $\dot{V}O_{2}$‐PO nonlinearity is largest in constant‐power exercise (6 min, 300 W) (+16%), lower in step‐incremental exercise (30 W/3 min) (+12%), in slow ramp‐incremental exercise (10 W/min) (+8%), in medium ramp‐incremental exercise (30 W/min) (+1%), and lowest (negative, downward deviation) in fast ramp‐incremental exercise (50 W/min) (−2%). The last theoretical result was caused by the fact that the increase in $\dot{V}O_{2}$ due to the increase in the additional ATP usage was smaller than the decrease in $\dot{V}O_{2}$ related to elevated ATP supply by anaerobic glycolysis. These theoretical predictions agree well, at least semiquantitatively, with experimental data concerning the pulmonary $\dot{V}O_{2}$‐PO nonlinearity in different exercise modes.

It was also postulated that while the kinetic properties of the system in voluntary constant‐power exercise can be satisfactorily accounted for by the saturating‐type *A*_OX_ (each‐step activation, ESA, intensity)‐*A*_UT_ (relative ATP usage activity) relationship, then the declining‐type *A*_OX_‐*A*_UT_ relationship seems to predict correctly the kinetic behavior of the system in step‐incremental exercise and ramp‐incremental exercise. For instance, the declining‐type *A*_OX_‐*A*_UT_ relationship is able to account for the significant increase in *t*_0.63_ (time to reach 63% of the $\dot{V}O_{2}$ amplitude during on‐transient, analogous to *τ*_p_) in step‐incremental exercise and the negative $\dot{V}O_{2}$‐PO nonlinearity in fast ramp‐incremental exercise (through stimulation of ATP supply by anaerobic glycolysis). Both phenomena were encountered in experimental studies. Therefore, it was proposed that the phenomenological (averaged over whole muscle) regulation of OXPHOS is different in constant‐power exercise, on the one hand, and in step‐ and ramp‐incremental exercise, on the other hand.

## Conflict of Interest

None declared.
